# Hydroxyapatite Reinforced Polyvinyl Alcohol/Polyvinyl Pyrrolidone Based Hydrogel for Cartilage Replacement

**DOI:** 10.3390/gels8090555

**Published:** 2022-09-01

**Authors:** Mallikarjun B. Jalageri, G. C. Mohan Kumar

**Affiliations:** Polymer Composites Laboratory, Department of Mechanical Engineering, National Institute of Technology Karnataka, Surathkal, Mangalore 575025, India

**Keywords:** biomaterial, ceramic, freeze-drying, polymer, tissue

## Abstract

Polyvinyl alcohol (PVA) and Polyvinyl Pyrrolidone (PVP) hydrogels are desirable biomaterials for soft tissue repair and replacement. However, the bio-inertness and poor cell adhesive potency of the PVA and PVP hinder the wide range of biomedical applications. In the present work, PVA and PVP were blended with a one-dimensional hydroxyapatite nanorod (HNr), and PVA/PVP/HNr composite hydrogel was synthesized by the freeze-thaw process. The developed hydrogels were characterized by Scanning Electron Microscope (SEM). The bio-ceramic nanohydroxyapatite content was optimized, and it was found that reinforcement improves mechanical strength as well as bioactivity. The compression strength values are 2.47 ± 0.73 MPa for the composite having 2 wt% of nanohydroxyapatite. The storage modulus was much higher than the loss modulus, which signifies the elastic dominancy similar to cartilage. Besides, the antimicrobial activity of nanohydroxyapatite reinforced PVA hydrogel towards bacterial species, *Escherichia coli* (*E. Coli*), *Staphylococcus aureus* (*S. aureus*) was satisfactory, and the in vitro biocompatibility response towards Human Mesenchymal stem cells(hMSC) after 72 h of culture confirms nanohydroxyapatite reinforced PVA/PVP hydrogels are the promising alternatives for next-generation cartilage substitutes.

## 1. Introduction

Hydrogels are a three-dimensional water-swollen polymeric material, and these hydrogels are the first biomaterials used for the human body [1]. In recent years, hydrogels utilization increased due to the applicability of hydrogels in biomedical developments and their unique ability to engineer materials that closely match natural tissues such as artificial cartilage [2], intraocular and contact lenses [3], nucleus pulposus [4] and soft actuators [5]. Hydrogels are also used in pharma industries as a drug delivery vehicle, wound dressing, care products, etc. [6]. Hydrogels can be developed from synthetic, natural, or hybrid polymers. The development of hydrogel involves Physical and chemical cross-linking. Crosslinking can be formed in different ways, including solution casting, solution mixing, and interpenetrating network formation [7]. ide reactions such as unreacted pendant groups and entanglement lead hydrogels to exert less mechanical stability and delayed response to external stimuli. Revolutionary ideas in designing hydrogels exhibit numerous opportunities within biomaterials research—new thought associated with the design of hydrogels that enhance mechanical stability and porosity. Recently, quite a few developments have widened the applicability of hydrogel materials [8]. Moreover, there are three major approaches to enhancing hydrogels’ mechanical stability and porosity, mainly by adding cross-linking agents, double network, or hybrid hydrogel synthesis, and reinforcing nanoparticles to the hydrogels.

Polyvinyl alcohol is a biocompatible, water-soluble polymeric synthetic material that has been used extensively in the fabrication of biomaterials due to its favorable properties such as nontoxicity, biocompatibility, and ease of fabrication [9]. PVA hydrogels synthesized via a freeze-thaw process and characterized to examine various physicochemical properties are compared to meniscal and artificial cartilage [10]. These biomaterials can mimic the function of human tissue. Polyvinyl pyrrolidone is a hydrogel with distinct properties such as good water solubility, nontoxicity, and high viscoelastic strength, which improves the mechanical properties of PVP composites, and good quality biocompatibility. Polyvinyl pyrrolidone is a membrane additive and pore former agent used in biomedical applications [11]. PVA hydrogel has been identified as a fascinating and promising material for articular cartilage replacement. The most significant limitation of PVA hydrogels is that their mechanical properties are difficult to match the requirements of articular cartilage [12].

Polyvinyl alcohol hydrogels are excellent biomaterials for cartilage scaffolds due to their structural similarities with biological tissues and inherent hydrophilicity [13]. However, their bioinertness and increased flexibility severely limit their applications to mechanical strength-required fields [14]. To improve performance, an appropriate modification is required. As a result, numerous efforts have been made to develop high-strength hydrogels. For example, consider double network hydrogels [15,16], hydrophobic associated hydrogels [17], ionically crosslinked hydrogels [18], and nanocomposite hydrogels [19]. Furthermore, various metallic and ceramic reinforcements such as silver [20], titanium dioxide [21], calcium phosphate [22], and zirconium [23,24] have been incorporated to create mechanically robust hydrogels. PVA/PVP blend hydrogels have been extensively studied as cartilage replacement materials. Functional compressive mechanics of a PVA/PVP nucleus pulposus replacement [4]. Embedding a small quantity of PVP molecules into the PVA improves network stability via interchain hydrogen bonding between both the PVA hydroxyl group and the PVP carbonyl groups, increasing crystallinity and decreasing PVA hydrogel degradation [25]. The mechanical and tribological properties of the PVP/PVA hydrogels were significantly dependent on the amount of PVP content, according to the results of the developed PVP/PVA composite hydrogel by repeated freeze and thaw process [26]. The inclusion of PVP into the PVA hydrogel enhances its mechanical properties and reduces the friction coefficient, which makes it to be one of the most promising alternatives for artificial cartilage [27].

Many nanohydroxyapatite syntheses are currently limited to chemical methods, which may impact cell proliferation in vivo. Furthermore, most nanohydroxyapatite research focuses on orthopedic and dental implant coating, with very few studies on the morphological influence of reinforcement on soft tissue scaffolds [28]. Optimizing the morphology and particle size of bioceramics used in biomedical applications is critical. Developing one-dimensional hydroxyapatite nanorods is an effective method for improving hydroxyapatite’s mechanical and biological properties. The polar modulus of elongated nanohydroxyapatite provides structural stability and a larger surface area in contact with osteoblasts. Furthermore, human bone is composed of rod-like nanohydroxyapatite with diameters of 20–50 nm and lengths of 100–300 nm. Under physiological conditions such as temperature, pH, and fluid composition, nano-hydroxyapatite is one of the most stable forms of calcium phosphate. It also has a larger surface area and high adversity, which aids in the adsorption and differentiation of osteoblast cells in a biological environment [29,30]. A thorough investigation has been made over calcium phosphate-based bioceramics due to their utilization in biomedical application (tissue engineering and pharmaceutical industries). Due to its favorable characteristics such as biocompatibility and osteoconductive, hydroxyapatite is regarded as the most promising biomaterial in orthopedic medicine. Its chemical composition is similar to that of natural bone [31].

Articular cartilage is a soft tissue layer covering diarthrodial joints’ surfaces. Cartilage damage caused by aging or physical injury leads to joint diseases such as osteoarthritis [32]. Its advanced composition and architecture allow it to withstand complex joint stresses. When cartilage is injured, it loses its ability to self-heal, which leads to osteoarthritis (OA), which causes severe pain and mobility loss [33]. Articular cartilage injury is a chronic and growing issue that affects millions of people around the world. Polyvinyl alcohol (PVA) biomaterials are promising transplants because they have properties similar to soft tissue; however, their low mechanical resistance and durability, as well as their inability to integrate with surrounding tissue, limit their application in this area [12]. Knee arthroplasty is a surgical procedure that uses cobalt-chromium alloys and high-density polyethylene to reconstruct a damaged knee. It has a few drawbacks, such as removing healthy bone during surgery for scaffold fixation, and if the first surgery fails, making the second attempt much more difficult. Nonetheless, cartilage regeneration is primarily intended to address three issues. Holds the compressive load first, then generates flow. Second, the reconstructed zone could induce stem cells in the subchondral bone. Third, the reconstructed zone promotes cartilage tissue regeneration by inducing the formation of osteoblasts, chondrocytes, and stromal cells from bone marrow [34,35]. There is an urgent need to create an advanced biomaterial that has the physical, mechanical, and biological properties of natural cartilage.

From our observation so far, very few reports were found on hydroxyapatite nanorod-reinforced PVA/PVP nanocomposite hydrogel for tissue-engineered cartilage scaffold. By taking into account, it was planned to synthesize hydroxyapatite nanorods using cuttlefish bone through a simple mechanochemical route. It was then blended with PVA/PVP to develop nanocomposite hydrogel using the freeze and thaw route. The main objective of this work was to investigate the influence of hydroxyapatite nanorods over the PVA/PVP-based double network hydrogel used to replace cartilage. The general idea behind this work is that the addition of phosphate group improves the mechanical property, bioactivity, and morphological characteristics of the PVA/PVP-based hydrogel.

## 2. Results and Discussion

### 2.1. Hydroxyapatite Nanorods Characterization

**Figure 1:**
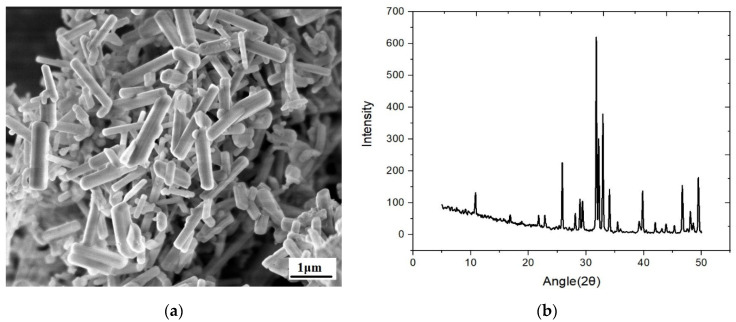
(**a**) SEM image of Hydroxyapatite nanorods, (**b**) X-ray diffraction spectrum.

The morphology of the hydroxyapatite nanorods was analyzed using FESEM. Figure 1a exhibits nanorod structure and spread in various arrangements and has a diameter ranging from 100 nm to 600 nm was analyzed using Image J software. XRD spectrum demonstrates a hexagonal crystal system, a common form of calcium phosphate. The hydroxyapatite phase was verified with JCPDS CARD:09-432, and significant peaks were observed between 20–35°, which indicates the better crystallinity of hydroxyapatite as shown in Figure 1b. 

### 2.2. Morphological Study of Nanocomposite Hydrogel

**Figure 2:**
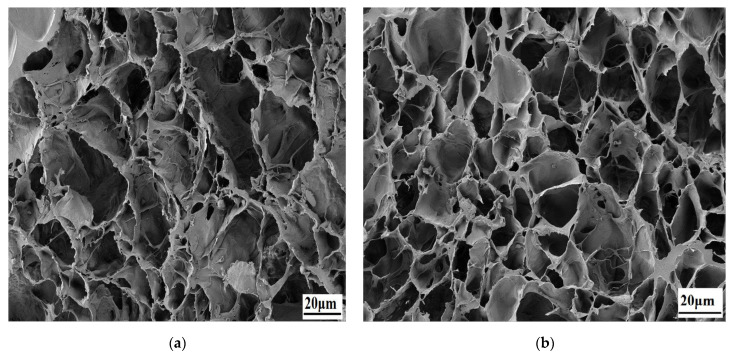
Scanning electron micrographs of (**a**) PVA/PVP and (**b**) PVA/PVP/2HNr hydrogel.

Figure 2 displays SEM images of the hydrogel scaffolds in a cross-sectional view. A porous structure with well-connected porosity is present in all lyophilized hydrogels, which is necessary for the cartilage scaffold to transport nutrients and metabolic wastes to the biological system. Table 1 provides an overview of the hydrogels’ average porosity. The average porosity of PVA/PVP scaffolds is 84.77 ± 0.41. Incorporating Inorganic hydroxyapatite causes the ordered microstructure and pore boundaries in the composite hydrogel to collapse and disappear. According to SEM pictures, adding hydroxyapatite to the PVA/PVP matrix transforms the microstructure from an undefined porous structure to a rounded porous structure. This porosity is beneficial for nutrient transfer and the extracellular matrix that provides for cell adhesion, cell-to-cell communication, and dedifferentiation. On the other hand, the structure, pore size, and porosity can be controlled.

### 2.3. Swelling Behaviour

**Figure 3:**
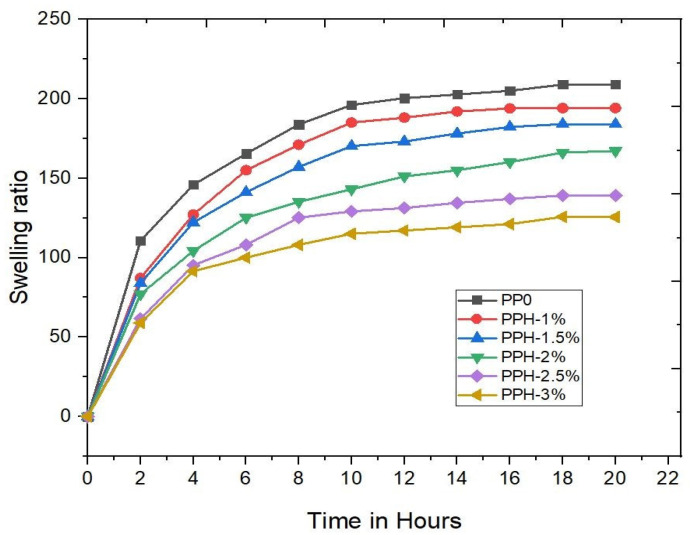
Swelling behaviors of PVA/PVP/HA hydrogels.

**Figure 4:**
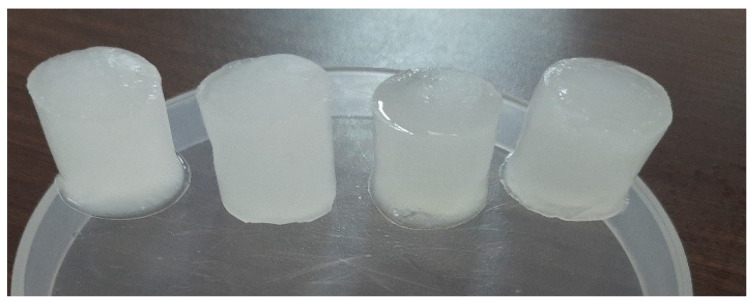
Macroscopic view of the swollen hydrogel.

Swelling studies are preliminary in vitro tests for the hydrogels; swelling studies are performed for PVA/PVP with different compositions of HNr reinforcement. Swelling strength is a very important metric for biomaterials in tissue engineering applications [36]. Pore size and interconnectivity play a key role in body fluid absorption, nutrient transport, and metabolite exchange [37]. Furthermore, swelling expands the pore volume, thus increasing the inner surface area/volume and increasing the likelihood of cell infusion from the surface to the interior. However, a higher swelling ratio negatively affects the mechanical stability of the scaffold. Composites with higher reinforcement concentrations have lesser swelling abilities, so the swelling strength gradually decreases, as shown in Figure 3. Previous research suggests that as reinforcement increases, water absorption will get decrease. This could be caused by secondary electrostatic bonds between the reinforcement and polymer structure [38]. We determined the porous structure of the scaffold with interconnected porosity using SEM analysis, as shown in Figure 2. The swollen hydrogel macroscopic image is shown in Figure 4. The abbreviations are used in the figures. PP0 denotes a combination of pure PVA/PVP similarly. PPH1%, PPH 1.5%, PPH2%, PPH 2.5%, PPH3% denotes PVA/PVP fortified with different concentrations of hydroxyapatite (1, 1.5, 2, 2.5, 3)%.

Similarly, surface wettability affects the protein, essential nutrient adsorption, and cell differentiation on the surface scaffold. Surface wetting characteristics of PVA/PVP/HA composites are examined by contact angle measurement. The contact angle data are summarised in Table 1, and the reduction of contact angles is observed as an increment in reinforcement from 0 to 3%. Pure PVA/PVP hydrogel exhibit a contact angle of 64.96 ± 0.94°. The surface hydrophilicity of composite hydrogel increases with increases in the concentration of HA.this result reveals the hydrophilic nature of the hydrogel.

### 2.4. Compression Strength

**Figure 5:**
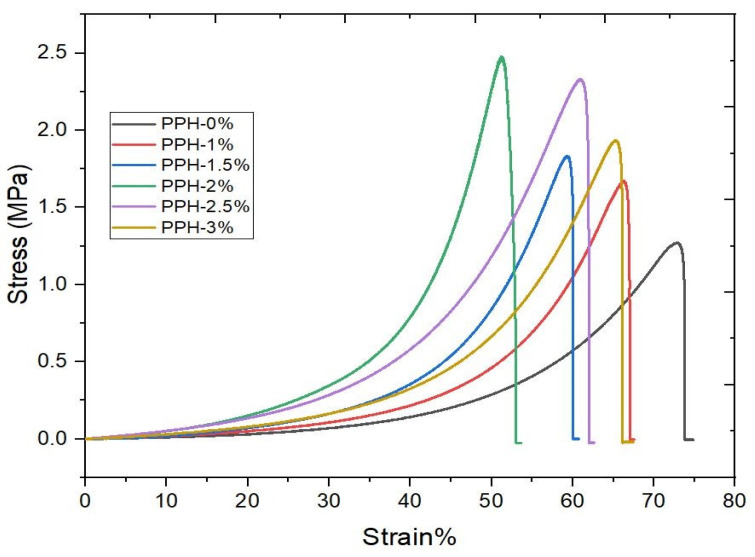
Mechanical characterization of composite hydrogels under unconfined compression.

The stress-strain graph of the hydrogel materials is depicted in Figure 5. the obtained graph corresponds to the nature of viscoelastic solids. The behavior of hydrogel under compression is strongly influenced by the polymer structure, which is similar to the cartilage under compression [38]. When a polymeric hydrogel is loaded, the load is absorbed, and the polymeric chains are reoriented, causing the interstitial fluid to flow out of the hydrogel.

During this, a load is sufficient for significant deformation. When the application of load continues, the reorientation leads to uniformity, and friction from interstitial fluids causes the gelly materials to harden and need extra effort to improve the additional strain. In the present data, adding 2 wt% of hydroxyapatite gives good compressive strength of (2.47 MPa), as shown in Figure 5. this result is matched with the compression strength of the healthy cartilage, which lies between 0.1 to 2 MPa [39]. Furthermore, hydrogels above 2%HA decrease compressive strength. This may be possible due to the agglomeration of hydroxyapatite nanoparticles. As the load is applied, the agglomeration point itself acts as a crack generator; as loading increases, the crack propagates and damages the hydrogel sample. All the hydrogels stress, strain and young’s modulus details are tabulated in Table 2.

### 2.5. Rheological Behavior of Hydrogel

**Figure 6:**
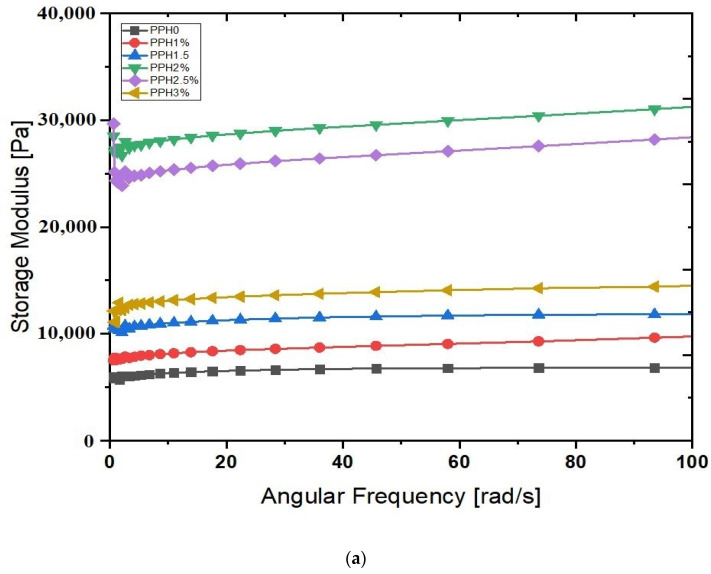

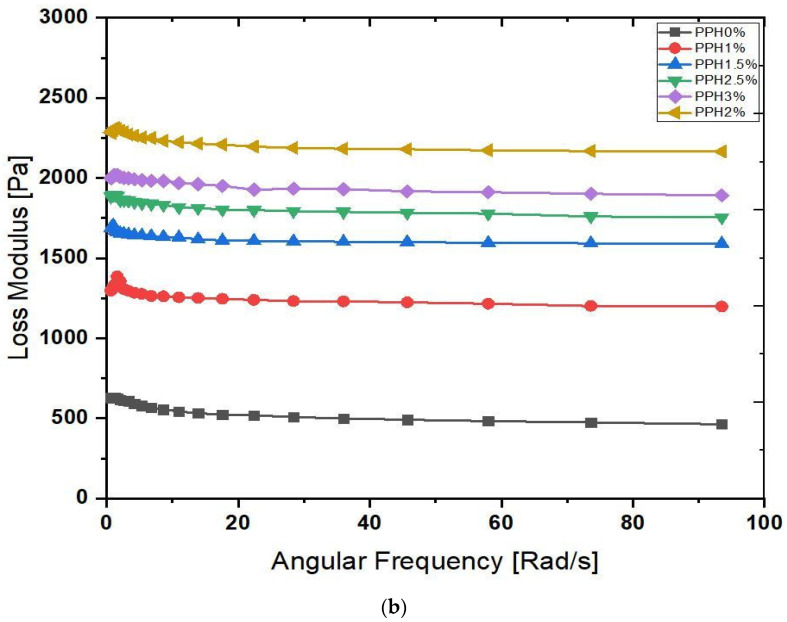
Rheological properties of PVA/PVP/HNr Composite hydrogel. (**a**) StorageModulus, (**b**) Loss modulus.

The viscoelastic nature of the hydrogels was investigated by oscillatory frequency sweep using MCR 702 Anton-Paar rheometer. The storage modulus (G′) and loss modulus (G″) of hydrogels with Nano hydroxyapatite mass fractions are shown in the figures. The G′ (storage modulus) represents hydrogel sample elasticity, defined as energy stored due to elasticity, and G″ represents hydrogel sample viscosity. From the figures, it was observed that the storage modulus of all the samples is not dependent on the frequency. Still, the increment followed by increases in mass fractions of hydroxyapatite nanoparticles until 2%Hap then decrement observed until 3%Hap is the loss modulus (G″) situation in Figure 6b. Pan et al. reported similar output, indicating that storage and loss modulus increased to a maximum value when nano-hydroxyapatite mass fraction was 6%, then decreased as the hydroxyapatite increased to 9% [40].

This phenomenon is subjected relationship between rheometer angular frequency and chain segment oscillation. Storage modulus is independent of the frequency, implying macromolecular chains can keep up with changes in angular frequency, and the effect of hysteresis is significantly less. The trend of increasing storage modulus with increasing hydroxyapatite amount suggests that hydroxyapatite can adapt hydrogel to higher movement frequency, which can be used in extreme conditions. When the hydrogel is loaded with external loads, the matrix transfers the load to the hydroxyapatite nanoparticle, increasing the hydrogel’s strength.

Figure 6a depicts the relationship between storage and loss modulus of PVA/PVP/HNr composites. The storage modulus (G′) for all hydrogels is always greater than the loss modulus (G″), indicating that composites are elastic in nature rather than in a fluid-like state. The elastic modulus of all composite hydrogels is greater than that of PVA/PVP. Demonstrates that the matrix and reinforcement have a good interaction. The fluid in the nanopores, on the other hand, interlocks the polymer network and provides mechanical stiffness to resist shear deformation.

### 2.6. Antimicrobial and Cytotoxic Assay

**Figure 7:**
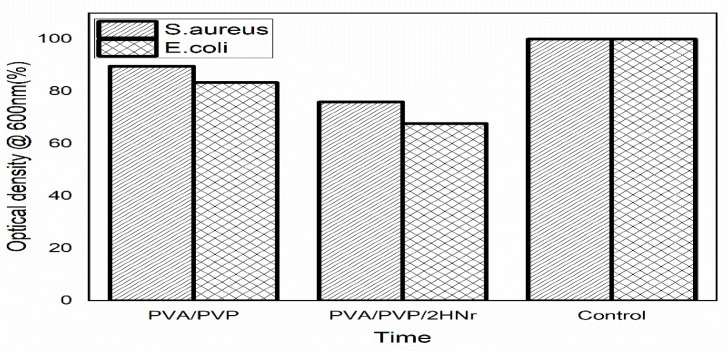
Antibiogram of PVA/PVP and PVA/PVP/2HNr hydrogel against Gram-negative *Escherichia coli*, Gram-positive *Staphylococcus aureus*.

The antimicrobial efficacy of bioactive material is crucial to resisting post-surgical infections and film formation. The growth inhibition of microbes on PVA/PVP and composite hydrogel are as shown in Figure 7. As expected, composite hydrogels PVA/PVP/2HNr show superior antimicrobial properties than the PVA/PVP hydrogel and they are more effective for *E. coli* than *S. aureus*, since 8% more inhibition was found for PVA/PVP/2HNr after 24 h of culture. This could be explained by the difference in the cellular cladding of Gram-negative and Gram-positive bacteria. Typically gram negative *E. coli* has a thin wall structure consisting of peptidoglycans and lipopolysaccharide, whereas Gram-positive bacteria such as *S. aureus* has a thick layer made of muco peptides murein and lipoteichoic acid, which resist the cytoplasmic leakage [41]. On the other hand, the Ca^2+^ ions of hydroxyapatite destabilize the cell membranes by reactive oxygen species (ROS) generation [42].

**Figure 8:**
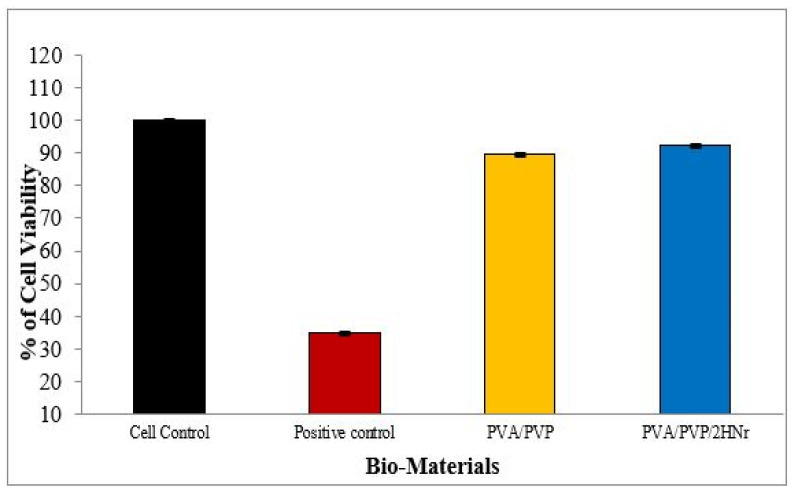
hMSC cell Viability of PVA/PVP and PVA/PVP/2HNr hydrogel after 72 h cell culture.

The in-vitro toxicity assay for the PVA/PVP-based composite hydrogel towards the hMSC Cell line was investigated by the MTT assay method. Compared to negative control (cell control) the cell viability of the positive control is three times the minimum as shown in Figure 8. Hydrogels PVA/PVP and PVA/PVP/2HNr exhibit 89.71% and 92.32% Cell viability, respectively, and little enhancement in the cell viability observed in the composite hydrogel (PVA/PVP/2HNr) compared to pure PVA/PVPMaybe this enhancement is due to releasing bioactive ions (Ca^2+^ and PO_4_^3−)^ from nanohydroxyapatite. The release of calcium and phosphate from nanohydroxyapatite increases the local concentration of Ca^2+^ and PO_4_^3−^ ions, thus stimulating bone mineral formation on the counter surface. In addition, it helps to adsorb the extracellular matrix proteins, thus enhancing cell proliferation.

**Figure 9:**
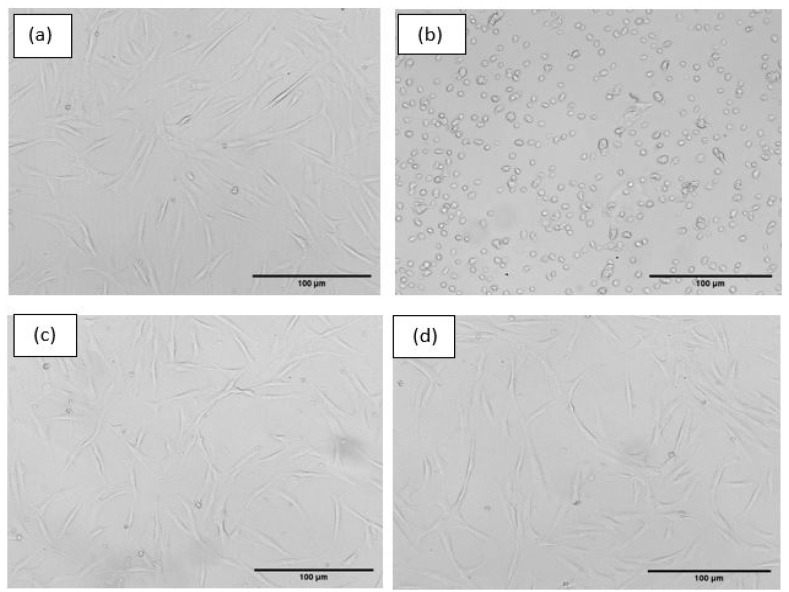
Inverted microscope images of hMSC Cells cultured for 72 h culture. (**a**) Untreated (**b**) Positive control, (**c**) PVA/PVP, and (**d**) PVA/PVP/2HNr.

According to standards of biocompatibility evaluation, composite hydrogel did not show any apparent toxicity to hMSC cells, and its cell viability is more than 70%. These cell lines exhibit fusiform morphology, as shown in Figure 9. All the cells are cultured and proliferated well in the culture plate after 72 h of incubation.

## 3. Conclusions

In the present research, cuttlefish bone-derived hydroxyapatite was developed and hydroxyapatite reinforced PVA/PVP-based double network hydrogels were processed and characterized successfully to utilize as an engineered cartilage bioactive material. The hydroxyapatite shows one-dimensional rod-like morphology with a diameter ranging from 100 nm to 600 nm. All the developed hydrogels showed porous structure with interconnected porosity, which is an important index for cartilage scaffold. The swelling strength of the hydrogel is better than articular cartilage and has good hydrophilic properties. An important improvement in the mechanical property such as compression was found for composites having 2 wt% of nanohydroxyapatite and above 2 wt% of hydroxyapatite compressive strength decreases; this may be due to the agglomeration effect. The storage modulus value was much higher than the loss modulus, and the storage modulus value increases with an increase in the concentration of hydroxyapatite, implying that composites are dominant elastic hydrogels similar to mammalian soft tissues. The antimicrobial efficiency of the composite hydrogel was satisfactory and is nontoxic toward hMSC human mesenchymal stem cells, exhibiting more than 75% of cell viability after 72 h of culture. Agreeing with the above results and discussions, it can be concluded that the PVA/PVP/2%HNr composites are an excellent option in all prospective physical and mechanobiological properties. That could be a good candidate for next-generation biomaterial for articular cartilage and tissue engineering applications.

## 4. Materials and Methods 

Polyvinyl alcohol hot water-soluble (Mw-60,000–125,000) was obtained from Himedia Ltd., Mumbai, India. Polyvinyl pyrrolidone K30 was obtained from Loba Chemie Pvt. Ltd., Mumbai, India. Cuttlefish bones were obtained from Blue Water Foods and Export Pvt. Ltd. in Mangalore, India, and orthophosphoric acid was obtained from India. The Human Mesenchymal Stem cells (Bone Marrow) cell line was obtained from Sigma Aldrich. All other chemicals and solvents were used as they were.

### 4.1. Hydroxyapatite Nano Rod Synthesis

The nanohydroxyapatite was prepared using cuttlefish bones. In brief, Cuttlefish bones were cleaned and boiled in water to remove fleshy residue attached over the surface and dried at 60 °C in an incubator for 10 h. Dried cuttlefish bones were crushed and powdered and sheived using a 100-micron siever 2 g of cuttlefish powder were weighed and added to 100 mL deionized water and stirred using a magnetic stirrer for 2 h at 60 °C. Orthophosphoric acid was added to uphold the stoichiometric ratio (Ca/P = 1.67). The stirring process continued for 10 h, and the obtained mixture was dried and calcined at 800 °C with a 5 °C/minute heating rate for 4 h [43]. Figure 1 shows the XRD and SEM of the hydroxyapatite. 

### 4.2. PVA/PVP/HA Nanorods Hydrogel Sample Preparation

Hydrogels are synthesized by dissolving PVA and PVP separately in deionized water and stirring the solution with a magnetic stirrer until a clear solution is obtained. Later, both polymer solutions are mixed and stirred at 90 °C for 2 h to mix homogeneously. Meanwhile, hydroxyapatite nanorods with different mass fractions (1, 1.5, 2, 2.5, and 3 wt% HNr) were homogeneously distributed using a sonicator. Then it was mixed with a blended solution of PVA/PVP, and the stirring process was continued for the next 10 h at 80 °C. The gel was transferred to a Petri dish, and for cross-linking, prepared samples were processed for five freeze and thaw cycles at −23 °C for 10 h, freezing, and 4 h thawing at room temperature to produce PVA/PVP/HA hydrogels. 

### 4.3. Morphological Analysis of PVA/PVP/Hap Nanorods Composite Hydrogel

Initially, hydrogels were lyophilized and fractured to study the hydrogel morphology. The cross-sectional section is used to determine the presence of porosity and the exact microstructure of the hydrogel. It is examined with a FESEM (HR-FESEM GEMINI 300, Carl Zeiss, Jena, Germany).

### 4.4. Swelling Studies 

For the swelling study, lyophilized hydrogels weights were measured and noted as Initial weight (*Wi*) before immersing them in deionized water, and later weight of the hydrogel was measured after dipping them into deionized water until its weight reached an equilibrium state and the weight of the sample was measured for every 2 h till it reaches an equilibrium state. The swelling ratio can be calculated by considering the initial weight and final weight ratio mentioned below in Equation (1). The porosity of the hydrogel was evaluated as presented elsewhere. In brief, the samples were immersed in ethanol for 12 h, and the weight was measured *(W*2).then, the samples were removed, and the initial weight (*W*1) was measured by lyophilizing the swollen hydrogel. The porosity was calculated by Equation (2).

(1) $$\mathrm{Swelling}\;\mathrm{rato}=\frac{\left(Wf-Wi\right)}{Wf}\;\times \;100$$

(2) $$\mathrm{Porosity}=\frac{\left(W2-W1-W3\right)}{\left(W2-W3\right)}\;\times \;100$$

where *W*3 is the weight of the ethanol remaining after removal of the swollen hydrogel sample.

### 4.5. Contact Angle Measurement

A contact angle analyzer was used to assess the wettability of the hydrogel samples, and 3 mm thick layered hydrogel samples were cast over the glass substrate to achieve flatness for the sample, which helps to measure the contact angle between the sample and droplet of the water. A liquid droplet was placed on a swollen flat hydrogel surface, and once the droplet reached an equilibrium, the contact angle was measured [44]. 

### 4.6. Compression Test

The unconfined compression strength was performed using a universal testing machine (Mec Mesin 10i–micro UTM) with 1 KN load cell, and the samples of size 10 mm in diameter and 7 mm in height were compressed up to 75% strain with the crosshead speed of 2 mm/min and stress vs. strain graph plotted for the test samples.

### 4.7. Rheological Study

The composite hydrogel was performed rheological testing at 37 °C using Anton and Par MCR 702 rheometer instrument. As well, an oscillatory frequency sweep analysis was performed at a frequency range of 0.1–100 Hz with a 1% fixed strain. The complex modulus of the components, such as storage and loss modulus, were investigated [45].

### 4.8. Antimicrobial Evaluation

The antimicrobial nature of the composite hydrogel against *E. coli* and *S. aureus* bacteria was evaluated by a growth inhibition assay. The bacterial culture was subcultured on a sterile broth, and the absorbance was measured (OD_i_). Then 10 mg of the hydrogel sample was added to the appropriate well and incubated at 37 °C. After 24 h of incubation, the optical density of the inoculated culture was monitored (OD_f_). The culture without the hydrogel was taken as a control, and the percentage inhibition of growth compared to the control was evaluated using Equation (3) [19].

(3) $$Percentage\;growth\;inhibition=\frac{ODf}{ODi}\times 100$$

### 4.9. Cytotoxicity Evaluation

The cytotoxicity of the gel sample was investigated by identifying the cell viability of the hMSC cell line using an MTT assay. In brief small 10 mm diameter discs 2 mm thick were sterilized using 70% ethanol and washed 3–4 times using water and PBS. Then samples were exposed to UV light for 20 min to ensure sterility of the sample. hMSC (Human Mesenchymal stem cells) 1000 μL of cell suspension containing 50,000 cells were seeded over the surface of the hydrogel and incubated on the plates for 72 h at 37 °C in 5% carbon dioxide media separately. Spent media was replaced by an MTT agent to the ultimate concentration of 0.5 mg/mL and incubated for a few hours. Reagents of MTT were removed, and add 100 μL of DMSO was gently stirred. The gyratory shaker will enhance the dissolution. In between, pipetting was done up and down to dissolve MTT formazan crystals in the dense culture. The cell viability was determined using the Equation (4) at 570 nm optical density.

(4) $$\%\;of\;Cell\;Viability=\frac{Mean\;OD\;of\;sample\;@\;570\;nm\;}{Mean\;OD\;of\;Control\;@\;570\;nm}\times 100\;$$

**Statistics:** All the quantitative results were obtained from three samples and data mean ± standard deviation. Statistical significance was calculated using the ANOVA Test. A value of *p* < 0.05 was considered as statistically significant.

## Figures and Tables

**Table 1:** PVA/PVP/HNr composite hydrogel physical properties.

Hydrogel	Swelling Ratio	Contact Angle (°)	Porosity (%)
PPH0%	209.03 ± 4.95	64.96 ± 0.94	84.77 ± 0.41
PPH1%	194.23 ± 4.45	60.25 ± 0.30	81.12 ± 0.24
PPH1.5%	184.07 ± 4.41	59.42 ± 0.79	76.12 ± 0.95
PPH2%	167.23 ± 4.97	53.77 ± 1.0	73.63 ± 0.51
PPH2.5%	139.16 ± 5.12	51.78 ± 1.4	71.12 ± 1.11
PPH3%	125.54 ± 3.13	48.60 ± 1.2	70.06 ± 0.41

**Table 2:** Properties of PVA/PVP/HNr hydrogel under unconfined compression.

Material Combination	Stress in MPa	Strain ε (%)	Modulus (E), in Mpa
PPH0%	1.27 ± 0.09	72.74 ± 0.44	0.24 ± 0.07
PPH1%	1.67 ± 0.15	66.19 ± 0.52	0.36 ± 0.06
PPH1.5%	1.83 ± 0.026	59.17 ± 1.83	0.44 ± 0.018
PPH2%	2.47 ± 0.73	51.11 ± 1.63	0.69 ± 0.034
PPH2.5%	2.33 ± 0.96	60.81 ± 1.39	0.54 ± 0.002
PPH3%	1.93 ± 0.74	65.15 ± 3.13	0.42 ± 0.003

## Data Availability

The datasets generated during the current study are available from the corresponding author on reasonable request.

## References

[1] Korah L.V., Anilkumar G., Thomas S. (2018). Hydrogels, DNA, and RNA polypeptides for the preparation of biomaterials. Fundamental Biomaterials: Polymers.

[2] Mantha S., Pillai S., Khayambashi P., Upadhyay A., Zhang Y. (2019). Smart Hydrogels in Tissue Engineering and Regenerative Medicine. Materials.

[3] Li L., Yu F., Zheng L., Wang R., Yan W., Wang Z., Xu J., Wu J., Shi D., Zhu L. (2019). Natural hydrogels for cartilage regeneration: Modification, preparation and application. J. Orthop. Transl..

[4] Joshi A., Fussell G., Thomas J., Hsuan A., Lowman A., Karduna A., Vresilovic E., Marcolongo M. (2006). Functional compressive mechanics of a PVA/PVP nucleus pulposus replacement. Biomaterials.

[5] Lee Y., Song W.J., Sun J.Y. (2020). Hydrogel soft robotics. Mater. Today Phys..

[6] Aswathy S.H., Narendrakumar U., Manjubala I. (2020). Commercial hydrogels for biomedical applications. Heliyon.

[7] Bashir S., Hina M., Iqbal J., Rajpar A.H., Mujtaba M.A., Alghamdi N.A., Wageh S., Ramesh K., Ramesh S. (2020). Fundamental concepts of hydrogels: Synthesis, properties, and their applications. Polymers.

[8] Kopecek J. (2007). Hydrogel biomaterials: A smart future?. Biomaterials.

[9] Teodorescu M., Bercea M., Morariu S. (2018). Biomaterials of Poly(vinyl alcohol) and Natural Polymers. Polym. Rev..

[10] Oliveira A.S., Seidi O., Ribeiro N., Colaço R., Serro A.P. (2019). Tribomechanical comparison between PVA hydrogels obtained using different processing conditions and human cartilage. Materials.

[11] Teodorescu M., Bercea M. (2015). Poly(vinylpyrrolidone)–A Versatile Polymer for Biomedical and Beyond Medical Applications. Polym. Plast. Technol. Eng..

[12] Gonzalez J.S., Alvarez V.A. (2014). Mechanical properties of polyvinylalcohol/hydroxyapatite cryogel as potential artificial cartilage. J. Mech. Behav. Biomed. Mater..

[13] Li F., Wang A., Wang C. (2016). Analysis of friction between articular cartilage and polyvinyl alcohol hydrogel artificial cartilage. J. Mater. Sci. Mater. Med..

[14] Chen K., Zhang D., Yang X., Cui X., Zhang X., Wang Q. (2016). Research on torsional friction behavior and fluid load support of PVA/HA composite hydrogel. J. Mech. Behav. Biomed. Mater..

[15] Yan X., Yang J., Chen F., Zhu L., Tang Z., Qin G., Chen Q., Chen G. (2018). Mechanical properties of gelatin/polyacrylamide/graphene oxide nanocomposite double-network hydrogels. Compos. Sci. Technol..

[16] Nurly H., Yan Q., Song B., Shi Y. (2019). Effect of carbon nanotubes reinforcement on the polyvinyl alcohol–polyethylene glycol double-network hydrogel composites: A general approach to shape memory and printability. Eur. Polym. J..

[17] Gao T.T., Niu N., Liu Y.D., Liu X.L., Gao G., Liu F.Q. (2016). Synthesis and characterization of hydrophobic association hydrogels with tunable mechanical strength. RSC Adv..

[18] Gierszewska M., Ostrowska-Czubenko J. (2016). Chitosan-based membranes with different ionic crosslinking density for pharmaceutical and industrial applications. Carbohydr. Polym..

[19] Wei Y., Chen K., Wu L. (2016). In situ synthesis of high swell ratio polyacrylic acid/silver nanocomposite hydrogels and their antimicrobial properties. J. Inorg. Biochem..

[20] Le Thi P., Lee Y., Thi T.T.H., Park K.M., Park K.D. (2018). Catechol-rich gelatin hydrogels in situ hybridizations with silver nanoparticles for enhanced antibacterial activity. Mater. Sci. Eng. C.

[21] Kumar N., Hazarika S.N., Limbu S., Boruah R., Deb P., Namsa N.D., Das S.K. (2015). Microporous and Mesoporous Materials Hydrothermal synthesis of anatase titanium dioxide mesoporous microspheres and their antimicrobial activity. Microporous Mesoporous Mater..

[22] Dorozhkin S. (2015). Calcium Orthophosphate-Containing Biocomposites and Hybrid Biomaterials for Biomedical Applications. J. Funct. Biomater..

[23] Plumlee K., Schwartz C.J. (2009). Improved wear resistance of orthopedic UHMWPE by reinforcement with zirconium particles. Wear.

[24] Jiang H., Zhang G., Feng X., Liu H., Li F., Wang M., Li H. (2017). Room-temperature self-healing tough nanocomposite hydrogel crosslinked by zirconium hydroxide nanoparticles. Compos. Sci. Technol..

[25] Thomas J., Lowman A., Marcolongo M. (2003). Novel associated hydrogels for nucleus pulposus replacement. J. Biomed. Mater Res. A..

[26] Kanca Y., Milner P., Dini D., Amis A.A. (2018). Tribological properties of PVA/PVP blend hydrogels against articular cartilage. J. Mech. Behav. Biomed. Mater..

[27] Ma R., Xiong D., Miao F., Zhang J., Peng Y. (2010). Friction properties of novel PVP/PVA blend hydrogels as artificial cartilage. J. Biomed. Mater. Res. A.

[28] Turnbull G., Clarke J., Picard F., Riches P., Jia L., Han F., Li B., Shu W. (2018). 3D bioactive composite scaffolds for bone tissue engineering. Bioact. Mater..

[29] NasiriTabrizi B., Fahami A., EbrahimiKahrizsangi R., Ebrahimi F. (2012). New Frontiers in Mechanosynthesis: Hydroxyapatite–and Fluorapatite–Based Nanocomposite Powders. Nanocomposites: New Trends and Developments.

[30] Daryan S.H., Javadpour J., Khavandi A., Erfan M. (2018). Morphological evolution on the surface of hydrothermally synthesized hydroxyapatite microspheres in the presence of EDTMP. Ceram. Int..

[31] Fiume E., Magnaterra G., Rahdar A., Verné E., Baino F. (2021). Hydroxyapatite for biomedical applications: A short overview. Ceramics.

[32] Patel K.D., Singh R.K., Lee J.H., Kim H.W. (2019). Electrophoretic coatings of hydroxyapatite with various nanocrystal shapes. Mater. Lett..

[33] Patel J.M., Saleh K.S., Burdick J.A., Mauk R.L. (2019). Bioactive Factors for Cartilage Repair and Regeneration:Improving Delivery, Retention, and Activity. Acta Biomater..

[34] Naahidi S., Jafari M., Logan M., Wang Y., Yuan Y., Bae H., Dixon B., Chen P. (2017). biocompatibility of hydrogel-based scaffolds for tissue engineering applications. Bio. Adv..

[35] Sinha A., Guha A. (2009). Biomimetic patterning of polymer hydrogels with hydroxyapatite nanoparticles. Mater. Sci. Eng. C.

[36] Santosh Kumar B.Y., Isloor A.M., Anil S., Venkatesan J., Kumar G.C.M. (2019). Calcium phosphate bioceramics with polyvinyl alcohol hydrogels for biomedical applications. Mater. Res. Express.

[37] Nikolova M.P., Chavali M.S. (2019). Recent advances in biomaterials for 3D scaffolds: A review. Bioact. Mater..

[38] Weizel A., Distler T., Schneidereit D., Friedrich O., Bräuer L., Paulsen F., Detsch R., Boccaccini A.R., Budday S., Seitz H. (2020). Complex mechanical behavior of human articular cartilage and hydrogels for cartilage repair. Acta Biomater..

[39] Pan Y., Xiong D., Gao F. (2008). Viscoelastic behavior of nano-hydroxyapatite reinforced poly(vinyl alcohol) gel biocomposites as an articular cartilage. J. Mater. Sci. Mater. Med..

[40] Mow V.C., Guo X.E. (2002). Mechano-electrochemical properties of articular cartilage: Their inhomogeneities and anisotropies. Annu. Rev. Biomed. Eng..

[41] Siripatrawan U., Kaewklin P. (2018). Food Hydrocolloids Fabrication and characterization of chitosan-titanium dioxide nanocomposite film as ethylene scavenging and antimicrobial active food packaging. Food Hydrocoll..

[42] Riaz M., Zia R., Ijaz A., Hussain T., Mohsin M., Malik A. (2018). Synthesis of monophasic Ag doped hydroxyapatite and evaluation of antibacterial activity. Mater. Sci. Eng. C.

[43] Chen F., Ni Y., Liu B., Zhou T., Yu C., Su Y., Zhu X., Yu X., Zhou Y. (2017). Self-crosslinking and injectable hyaluronic acid/RGD-functionalized pectin hydrogel for cartilage tissue engineering. Carbohydr. Polym..

[44] Kumar B.Y., Isloor A.M., Kumar G.C., Asiri A.M. (2019). Nanohydroxyapatite Reinforced Chitosan Composite Hydrogel with Tunable Mechanical and Biological Properties for Cartilage Regeneration. Sci. Rep..

[45] Al-saud L.M. (2021). Comparative evaluation of Rheological characteristics of Giomers and other Nano-flowable resin composites in vitro. Biomater. Investig. Dent..

